# Transcript‐Level Modulation of *O*‐GlcNAc Transferase for Aging‐Related Neurodegenerative Diseases

**DOI:** 10.1002/cbic.202500774

**Published:** 2026-02-02

**Authors:** Florian Malard

**Affiliations:** ^1^ Univ. Bordeaux CNRS INSERM ARNA UMR 5320, U1212 Bordeaux France; ^2^ Univ. Bordeaux CNRS INSERM IECB US1 UAR 3033 Pessac France

**Keywords:** alternative splicing, antisense oligonucleotide, neurodegenerative diseases, *O*‐GlcNAcylation, *O*‐GlcNAc transferase, RNA therapeutics

## Abstract

The *O*‐GlcNAc Transferase (OGT) is responsible for the addition of β‐*O*‐linked N‐acetyl‐D‐glucosamine (*O*‐GlcNAc) to serine and threonine residues, thereby regulating more than 8000 human proteins through *O*‐GlcNAcylation. In the brain, reduced *O*‐GlcNAc levels, which can arise from insufficient OGT activity, have been increasingly linked to aging‐related neurodegenerative diseases such as Alzheimer's, Parkinson's, and amyotrophic lateral sclerosis. While current strategies focus on restoring *O*‐GlcNAc levels via *O*‐GlcNAcase (OGA) inhibition, recent discoveries highlight transcript‐level regulation of *OGT* as a direct and promising therapeutic target. This concept article explores the role of intron detention and decoy exon‐mediated splicing repression in limiting *OGT* pre‐mRNA maturation and proposes the use of antisense oligonucleotides or selective splicing factor degraders to promote productive splicing and nuclear export of *OGT* mRNA. By enhancing *OGT* expression independently of *O*‐GlcNAc feedback, these approaches aim to restore proteostasis and improve resilience to neurodegeneration, offering a novel therapeutic approach for aging‐related neurodegenerative diseases.

## Graphical abstract

*O*‐GlcNAc Transferase (OGT) and *O*‐GlcNAcase (OGA) maintain *O*‐GlcNAc homeostasis, but *OGT* expression and activity decline in the aging brain, promoting protein aggregation and neurodegeneration. Current approaches raise *O*‐GlcNAc levels via OGA inhibition. New insights into *OGT* splicing enable direct *OGT* upregulation using ASO‐ or PROTAC‐based strategies, alone or with OGA inhibition, potentially improving control of *O*‐GlcNAc homeostasis and therapeutic outcomes.

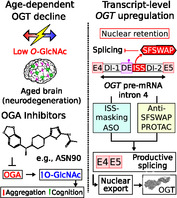

## 1 Introduction

**FIGURE 1:**
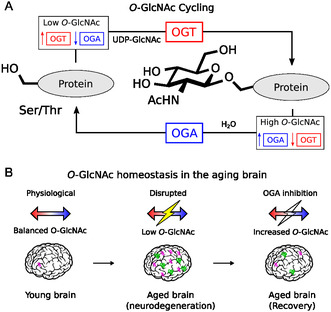
*O*‐GlcNAc dynamics. (A) *O*‐GlcNAc cycling. The *O*‐GlcNAc Transferase (OGT) adds β‐*O*‐linked N‐acetyl‐D‐glucosamine (*O*‐GlcNAc) to serine/threonine residues, and the *O*‐GlcNAcase (OGA) removes it [2]. *OGT* and *OGA* expression is controlled by *O*‐GlcNAc levels in a nutrient‐sensitive manner to maintain physiological protein *O*‐GlcNAcylation. (B) *O*‐GlcNAc homeostasis in the aging brain. Younger brains maintain physiological *O*‐GlcNAc balance, aged brains undergoing neurodegeneration can show disrupted regulation with low *O*‐GlcNAc and abnormal protein assemblies. Symptoms of neurodegeneration can be attenuated through OGA inhibition [3], restoring *O*‐GlcNAc homeostasis.

The *O*‐GlcNAc transferase (OGT) is responsible for adding *β*‐*O*‐linked N‐acetyl‐D‐glucosamine (*O*‐GlcNAc) to serine and threonine residues of cytosolic, nuclear, and mitochondrial proteins, modifying more than 8000 proteins in the human proteome [1]. Protein *O*‐GlcNAcylation is reversed by the *O*‐GlcNAcase (OGA), which removes the modification in a dynamic process called the *O*‐GlcNAc cycling [2] (Figure 1A). Within the cell, the *O*‐GlcNAcylation reaction catalyzed by OGT serves as an essential regulatory mechanism for transcription, signal transduction, stress responses, and proteostasis [4, 5, 6, 7]. Unlike other glycosylation pathways, *O*‐GlcNAcylation does not extend into complex structural motifs but instead acts as a nutrient‐sensitive signaling mechanism dependent on the metabolic state of the cell [8]. Importantly, disruption of protein *O*‐GlcNAcylation directly interferes with protein phosphorylation status, as both modifications often compete through increasingly well‐characterized crosstalk [9].

The *OGT* gene is located on the X chromosome and exhibits complex, multilevel regulation that controls both *OGT* expression and isoform balance. Indeed, the *OGT* gene gives rise to three major isoforms: nucleocytoplasmic OGT (ncOGT), mitochondrial OGT (mOGT), and small OGT (sOGT) [10]. While all three share an identical C‐terminal catalytic domain, the topology of these isoforms differs primarily in the number of N‐terminal tetratricopeptide repeats, which drive isoform‐specific substrate selectivity. Interestingly, alternative splicing was recently shown to be responsible for the nuclear retention of the full‐length *OGT* pre‐mRNA through a mechanism involving detained introns and a decoy exon [11, 12], which will be discussed further. While the tight regulation of *OGT* expression directly influences *O*‐GlcNAcylation dynamics with implications for disease, it is especially critical in the brain, where aging‐related declines in OGT activity can have important consequences for neuronal function and susceptibility to neurodegeneration [13].

## 2 OGT Activity in Aging‐Related Neurodegenerative Diseases

In the aging brain, the decline of *OGT* expression and activity contributes to a global reduction in protein *O*‐GlcNAcylation [3, 13]. Decreased *O*‐GlcNAc levels correlate with impaired cognitive function, diminished adult neurogenesis, and increased gliogenesis [14, 15]. The reduction in OGT‐mediated protein modifications disrupts the balance between *O*‐GlcNAcylation and phosphorylation, since both modifications often target the same or adjacent residues on key regulatory proteins [9]. This reciprocal relationship is critical in the context of neurodegeneration, where reduced *O*‐GlcNAcylation can lead to nonphysiological phosphorylation, cytoskeletal disorganization, and the formation of pathological protein aggregates [13].

In mice, the loss of *O*‐GlcNAc modification in forebrain excitatory neurons through *OGT* knockout induces neurodegeneration, including pathogenic processing of tau and amyloid precursor protein, widespread neuronal death, gliosis, and memory loss [16]. In mouse models of Alzheimer's disease (AD), enhancing OGT activity or inhibiting OGA has been shown to increase tau *O*‐GlcNAcylation, reduce tau hyperphosphorylation and neurofibrillary tangle formation, and lower amyloid‐*β* plaque formation, attenuate neuroinflammation, and improve cognitive functions [17, 18, 19, 20, 21]. Similarly, in Parkinson's disease (PD), experiments in mice and cultured cells have demonstrated that increased *O*‐GlcNAcylation leads to a reduction in *α*‐synuclein aggregation and dopaminergic neuron death, along with restoring dopamine release and motor function [22, 23, 24]. Finally, in amyotrophic lateral sclerosis (ALS), studies in mice and rats have shown that decreased *O*‐GlcNAcylation is associated with a reduced number of motor neurons, elevated reactive oxygen species, motor neuron death, and neurofilament loss, while upregulation of *OGT* expression in cultured cells attenuates TDP‐43 aggregation and reduces cellular toxicity [25, 26, 27, 28]. It is also worth noting that although *OGT* is expressed ubiquitously, *OGT* mRNA levels are higher in the brain than in other organs [29, 30], and cytosolic OGT activity is approximately ten times greater in the brain compared to muscle, adipose tissue, heart, and liver [31], making the brain one of the most glycosylated organs. In line with this, some cases of AD are increasingly referred to as type 3 diabetes, a condition that selectively affects the brain [32]. In this context, brain‐specific insulin resistance and impaired glucose metabolism were linked to both the processing of the amyloid pre‐cursor protein and the clearance of amyloid‐beta (A*β*) [33], potentially contributing to the initiation of AD pathology.

Altogether, these findings highlight the importance of OGT as a neuroprotective enzyme that maintains proteostasis, regulates phosphorylation networks, and prevents the accumulation of toxic protein aggregates. Therefore, modulating the expression or activity of OGT represents a promising therapeutic strategy for multiple aging‐related neurodegenerative diseases.

## 3 Current Approaches in *O*‐GlcNAc Modulation

As outlined in the previous section, *OGT* expression and activity tend to decrease in the aging brain, where this decline correlates with hallmark features of many aging‐related neurodegenerative diseases [34]. The direct upregulation of OGT activity using small molecules remains a significant challenge. Current pharmacological strategies instead rely on the inhibition of OGA, the enzyme that removes the *O*‐GlcNAc modification from protein residues [3] (Figure 1B).

**FIGURE 2:**
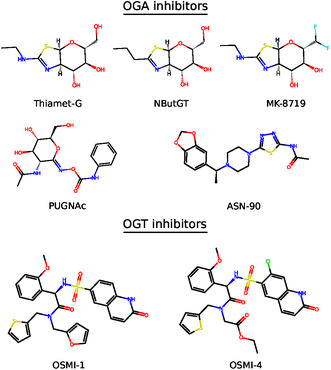
Representative chemical structures of known OGA and OGT inhibitors. The top panel shows examples of OGA inhibitors, including Thiamet‐G, NButGT, MK‐8719, PUGNAc, and ASN‐90. The bottom panel shows examples of OGT inhibitors, including OSMI‐1 and OSMI‐4.

The inhibition of OGA has demonstrated potent neuroprotective effects in various disease models by functionally mimicking OGT upregulation. Thiamet‐G, a widely used OGA inhibitor (Figure 2), has been shown to reduce tau hyperphosphorylation at key pathological residues in tauopathy models, including PC‐12 cells and the JNPL3 transgenic mouse model of AD, resulting in attenuated neurofibrillary tangle formation and neuronal loss [18]. In 5xFAD mice, a widely used model of amyloid pathology, treatment with the OGA inhibitor NButGT (Figure 2) reduced *γ*‐secretase activity via *O*‐GlcNAcylation of nicastrin, a core subunit of the *γ*‐secretase complex [19, 35]. This led to reduced A*β* production and plaque deposition, ultimately improving memory and reducing neuroinflammation [19, 35]. In genetically modified 5xFAD mice with reduced *OGA* expression, cognitive function and synaptic health were restored, indicating that long‐term elevation of *O*‐GlcNAcylation does not impair neuronal integrity and may be well tolerated [21, 23]. In PD models, elevation of *O*‐GlcNAc through thiamet‐G treatment reduced the aggregation of *α*‐synuclein and preserved dopaminergic neuron survival, resulting in improved dopamine release and partial recovery of motor function [36]. In ALS models, pharmacological inhibition of OGA has been shown to reduce oxidative stress and neuroinflammation in spinal motor neurons [27]. Furthermore, TDP‐43 aggregation was suppressed by increased *O*‐GlcNAcylation via *OGT* overexpression or GlcNAc supplementation, reducing cytotoxicity and inclusion body formation in neuronal cultures [28]. The clinical translation of OGA inhibition is underway, with inhibitors such as MK‐8719, ASN90, and LY3372689 currently undergoing clinical trials for tauopathies, including AD (Figure 2) [3, 37].

Although increasing *O*‐GlcNAc levels through OGA inhibition holds promise to treat aging‐related neurodegenerative diseases, the direct activation of OGT through transcript‐level interventions could represent an alternative or complementary approach that deserves further investigation.

## 4 Transcript‐Level Regulation of *OGT* Expression

**FIGURE 3:**
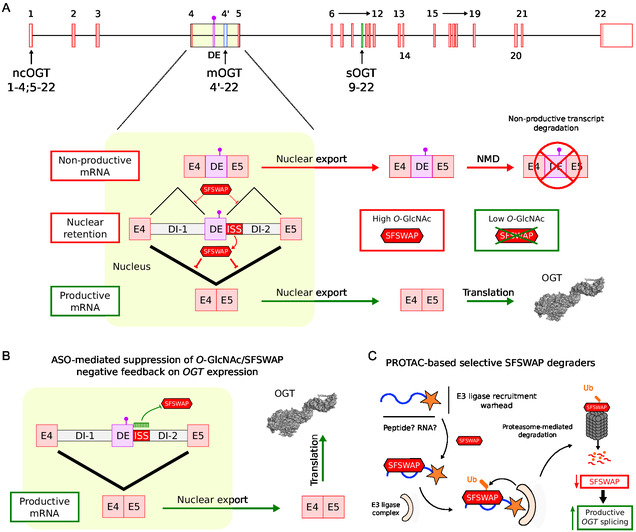
Transcript‐level regulation of *OGT* expression. (A) The *OGT* pre‐mRNA contains a detained intron (DI‐1, DI‐2)/decoy exon (DE) system within intron 4 that is responsible for nuclear retention of the transcript [9, 10]. Under high *O*‐GlcNAc levels, SFSWAP represses splicing of intron 4 in *OGT* pre‐mRNA through an ISS located downstream of the DE. In these conditions, residual splicing favors inclusion of the DE, leading to mRNA degradation via the NMD pathway and providing an additional regulatory layer. Under low *O*‐GlcNAc conditions, downregulation of SFSWAP promotes productive splicing of intron 4 and expression of functional OGT. (B) When *OGT* expression is repressed due to dysregulated *O*‐GlcNAc cycling, the negative feedback mechanism can be overridden by using a well‐designed ASO to mask the ISS sequence responsible for SFSWAP‐dependent repression of *OGT* pre‐mRNA splicing. This intervention restores *OGT* expression and increases *O*‐GlcNAc levels. (C) If SFSWAP binds directly to the ISS on *OGT* pre‐mRNA or indirectly through a protein partner, a selective SFSWAP degrader could be designed using an RNA or peptide moiety coupled with an E3 ligase recruitment warhead. This strategy would promote productive splicing of *OGT* pre‐mRNA and restore *O*‐GlcNAc levels.

*O*‐GlcNAc levels regulate so‐called detained intron splicing, a particular form of alternative splicing that leads to the nuclear retention of unspliced transcripts [38, 39]. While *O*‐GlcNAc controls detained intron splicing to tune system‐wide gene expression in response to nutrient conditions, it also specifically regulates *O*‐GlcNAc cycling enzymes (OGT and OGA) at the post‐transcriptional level [38]. Indeed, the regulation of *OGT* expression is tightly controlled at the transcript level through a mechanism involving the detention of intron 4 of *OGT* pre‐mRNA (Figure 3A), thereby modulating the cytoplasmic availability of mature *OGT* mRNA in response to cellular metabolic status [11]. Under conditions of high intracellular *O*‐GlcNAc, such as after treatment with the OGA inhibitor thiamet‐G, intron 4 of the *OGT* transcript is preferentially retained, leading to reduced *OGT* expression. Conversely, low *O*‐GlcNAc levels, such as those induced by the OGT inhibitor OSMI‐1 (Figure 2) or glucose deprivation, promote efficient splicing and removal of intron 4, leading to increased levels of cytoplasmic *OGT* mRNA and upregulation of *OGT* expression.

Key *cis*‐regulatory elements within intron 4 include an unannotated decoy exon, which partially overlap with an intronic splicing silencer (ISS), both playing crucial roles in promoting intron detention [11, 40]. Decoy exons are often found within large introns, and unproductive splicing complexes assembled at these exons lead to inefficient splicing and intron retention [40, 41]. In the case of the *OGT* decoy exon, it also contains a premature termination codon, which leads to nonsense‐mediated decay (NMD) of the transcript if the exon is spliced in. Therefore, the *OGT* decoy exon may also be referred to as a poison exon in this context [38]. The splicing factor suppressor of white apricot protein homolog (SFSWAP) was recently identified via a CRISPR knockout screen as a negative regulator of *OGT* intron 4 splicing [12]. Knockdown of SFSWAP results in enhanced splicing of the detained intron, especially under high *O*‐GlcNAc conditions, indicating that SFSWAP promotes intron detention. Should splicing still occur under negative SFSWAP regulation, it will favor the inclusion of the decoy exon that contains a premature termination codon, leading to transcript degradation through the NMD pathway. Upon low *O*‐GlcNAc or SFSWAP depletion, the productive splicing of *OGT* intron 4 is promoted, hence leading to the upregulation of *OGT* expression and the elevation of *O*‐GlcNAc levels. Interestingly, deletion of the *OGT* decoy exon/ISS or even blocking using morpholino oligonucleotides renders splicing of intron 4 constitutive [11, 42]. Although increased intron retention has been proposed as a post‐transcriptional signature associated with progressive aging and AD [43], further work is needed to determine whether this also correlates with increased intron retention in *OGT* pre‐mRNA.

Overall, the transcript‐level regulation of *OGT* consists of an autoregulatory feedback loop, where elevated OGT activity reduces the productive splicing of its own pre‐mRNA through SFSWAP‐dependent repression. This intron detention mechanism allows cells to fine‐tune OGT abundance in response to metabolic conditions, linking nutrient sensing with RNA processing.

## 5 Transcript‐Level Approaches for Therapeutic OGT Modulation

Current therapeutic strategies to modulate *O*‐GlcNAc in the aging brain focus on inhibiting OGA with small molecules, several of which are in clinical trials for tauopathies [3]. This emphasis reflects the challenge of developing OGT activators. However, recent insights into transcript‐level regulation suggest it may now be feasible to design therapeutics that bypass *O*‐GlcNAc‐dependent splicing control to promote constitutive *OGT* expression.

Among transcript‐level approaches, antisense oligonucleotides (ASOs) represent a viable and robust strategy (Figure 3B). The SFSWAP‐dependent repression of *OGT* pre‐mRNA splicing enables cells to modulate *OGT* expression in response to intracellular *O*‐GlcNAc levels, forming a homeostatic feedback loop that becomes disrupted in aging‐related neurodegenerative diseases. To override this mechanism, splice‐switching or steric‐blocking ASOs could be designed to target the intron 4 in *OGT* pre‐mRNA, masking either the splice sites surrounding the decoy exon or the overlapping ISS that mediate negative splicing regulation by SFSWAP. Such a strategy would promote productive splicing, leading to nuclear export of the mature *OGT* mRNA and an increase in OGT protein and *O*‐GlcNAc levels. Notably, ASO‐mediated upregulation of *OGT* expression would occur even under repressive *O*‐GlcNAc conditions that would normally suppress it, thereby bypassing the endogenous repression circuit and offering a means to directly increase OGT in disease‐relevant tissues, such as the aging brain. In this context, ASOs provide a wide range of chemical modifications (e.g., phosphorothioate backbones, 2^′^‐MOE) and conjugation strategies (e.g., GalNAc, peptide‐based carriers) to optimize brain delivery, some of which are already used in FDA‐approved therapies targeting the central nervous system [44].

As an alternative approach, proteolysis‐targeting chimeras (PROTACs) could be used to promote the productive splicing of *OGT* pre‐mRNA by selectively degrading relevant splicing factors, such as SFSWAP (Figure 3C). Strictly speaking, PROTACs were originally defined as bifunctional small molecules that bind specifically to a target protein while recruiting an E3 ligase complex, thereby triggering selective protein degradation via the proteasome pathway [45]. The concept has since evolved, with heterogeneous modalities such as peptide‐PROTACs and RNA‐PROTACs being developed [46, 47]. Herein, these variants can consist of a peptide or RNA moiety that binds specifically to the target protein, coupled with a small molecule or peptide that recruits an E3 ligase complex. In this context, it remains unclear if SFSWAP binds directly to the ISS region of intron 4 in *OGT* pre‐mRNA, while it is known that SFSWAP uses its SURP domain to interact with the splicing factor SF1 [48]. Further in vitro studies could investigate SFSWAP binding to RNA or identify the minimal SF1‐derived peptide capable of interacting with SFSWAP, thereby enabling the design of peptide‐ or RNA‐based PROTACs that selectively degrade SFSWAP and promote productive splicing of *OGT* pre‐mRNA, even under repressive *O*‐GlcNAc conditions that would otherwise suppress it. Targeted degradation of SFSWAP would mimic the effects of a knockdown in a reversible and specific manner, offering a strategy to restore OGT levels in disease states without directly targeting RNA sequences or relying on broad OGA inhibition, though with drawbacks discussed further.

Although direct small‐molecule OGT activators remain elusive, small‐molecule OGA inhibitors could still be combined with transcript‐level modulation of *OGT* expression to prevent potential positive feedback in *OGA* expression upon OGT activation, thereby enabling end‐to‐end control of *O*‐GlcNAc cycling and homeostasis.

## 6 Challenges and Limitations

Transcript‐level regulation of *OGT* is a promising strategy to elevate *O*‐GlcNAc levels in aging‐related neurodegenerative diseases, although several challenges and limitations can be anticipated.

From a mechanistic perspective, the approach involves overriding the negative feedback loop sensitive to *O*‐GlcNAc levels that regulates *OGT* splicing and expression. Bypassing this mechanism could help counteract insufficient *O*‐GlcNAc levels in the aging brain or the negative feedback on *OGT* expression triggered by OGA inhibition. However, the potential for sustained *OGT* overexpression may lead to excessive *O*‐GlcNAcylation, potentially disrupting transcriptional regulation and phosphorylation‐dependent signaling. These concerns appear less problematic in the context of the aging brain, where elevated *O*‐GlcNAc levels through OGA inhibition seem to be well tolerated in disease models [21, 23], reinforcing the relevance of this specific setting. Additional mechanistic studies could investigate the precise causes of decreased *OGT* expression in the aging brain and its contribution to the initiation and progression of neurodegeneration. This decrease in *OGT* expression may result from multiple factors, including age‐dependent disruptions in *OGT* intron 4 splicing, transcriptional regulation, differential isoform expression, cellular metabolism, or other mechanisms [14, 49, 50, 51].

An additional layer of complexity in transcript‐level *OGT* modulation is related to its known isoforms: ncOGT, mOGT, and sOGT. It remains unclear how these isoforms arise from the single *OGT* gene, with reports suggesting that alternative splicing or alternative start sites may be responsible for the minor mitochondrial and small OGT isoforms [10, 52, 53]. In the alternative splicing scenario, transcript‐level modulation of *OGT* may apply only to the major nucleocytoplasmic isoform, thereby altering the cellular balance of OGT isoforms. In the alternative start site scenario, it could regulate global OGT levels while leaving the isoform balance unaffected. Although ncOGT overexpression has been shown to be nontoxic to cells, overexpression of mOGT has been associated with mitochondrial dysfunction and cellular apoptosis [54]. Dysregulation of mOGT has also been proposed to contribute to the onset of AD [55]. Therefore, care should be taken in developing transcript‐level modulators of *OGT* to monitor their impact not only on ncOGT but also on mOGT expression and related toxicity. Overall, manipulation of OGT isoform balance remains a prospective goal given the current state of the field, and further investigations are needed to achieve comprehensive isoform‐specific control of OGT.

Among the proposed pharmacological approaches to increase OGT activity at the transcript level, ASOs offer the advantage of high sequence specificity, though they carry the risk of off‐target effects due to potentially degenerate splicing silencer target sequences, which may modulate genes unrelated to *OGT*. Therefore, the transcriptome‐wide evaluation of *OGT*‐directed ASOs will be required before considering therapeutic applications. Regarding PROTAC‐based degraders of SFSWAP, selective degradation of this splicing factor could globally disrupt intron detention, as SFSWAP has been characterized as a master regulator of this process [12]. If global intron detention is correlated with increased intron retention during aging [43], this major drawback might be partially mitigated. At this point, it can still be noted that knockdown of SFSWAP using siRNA does not appear to trigger immediate toxicity in cells [12]. Nonetheless, evaluating transcript‐level modulation of *OGT* expression using ASOs or PROTACs in relevant disease models will be essential to determine whether to pursue this therapeutic approach as is, to combine it with known OGA inhibitors, or to develop small‐molecule splicing modifiers, which may be more suitable for therapy but will require considerably more complex design.

Overall, the current state of the art readily enables studies on transcript‐level modulation of *OGT* in cell models of aging‐related neurodegenerative disease. Further preclinical investigations using ASOs or PROTACs will need to draw inspiration from recent developments to achieve efficient drug delivery to the brain. Nevertheless, the progress of OGA inhibitors in clinical trials for aging‐related neurodegenerative disease strongly justifies exploring transcript‐level modulation of *OGT* as an alternative or complementary approach.

## 7 Conclusion

The transcript‐level regulation of *OGT* is proposed within the context of aging‐related neurodegenerative diseases. In light of recent clinical developments involving small molecules that upregulate *O*‐GlcNAc levels through OGA inhibition [3], this transcript‐level strategy aims to directly promote *OGT* expression by disrupting the autoregulatory mechanism responsible for *OGT* pre‐mRNA retention in the nucleus [12]. This approach seeks to increase OGT levels independently of the negative feedback exerted by elevated *O*‐GlcNAc levels on *OGT* expression. If feasible in the short to mid‐term, it could serve as an alternative or complementary strategy to OGA inhibition. A comparative evaluation of the biological efficacy of both approaches, individually or in combination, will be critical for selecting the most appropriate intervention based on specific disease and model contexts.

## Conflicts of Interest

The author declares no conflicts of interest.
